# A prospective study of the relationships between movement and glycemic control during day and night in pregnancy

**DOI:** 10.1038/s41598-021-03257-0

**Published:** 2021-12-13

**Authors:** Masoud Behravesh, Juan Fernandez-Tajes, Angela C. Estampador, Tibor V. Varga, Ómar S. Gunnarsson, Helena Strevens, Simon Timpka, Paul W. Franks

**Affiliations:** 1 Genetic & Molecular Epidemiology Unit, Department of Clinical Sciences, Lund University, Jan Waldenströms gata 35 214 28, Malmö, Sweden; 2 Section of Epidemiology, Department of Public Health, University of Copenhagen, Copenhagen, Denmark; 3 Department of Obstetrics and Gynecology, Skåne University Hospital, Malmö and Lund, Sweden; 4 Perinatal and Cardiovascular Epidemiology, Department of Clinical Sciences, Lund University, Malmö, Sweden; 5 Harvard TH Chan School of Public Health, Boston, MA USA

**Keywords:** Endocrine system and metabolic diseases, Epidemiology

## Abstract

Both disturbed sleep and lack of exercise can disrupt metabolism in pregnancy. Accelerometery was used to objectively assess movement during waking (physical activity) and movement during sleeping (sleep disturbance) periods and evaluated relationships with continuous blood glucose variation during pregnancy. Data was analysed prospectively. 15-women without pre-existing diabetes mellitus wore continuous glucose monitors and triaxial accelerometers from February through June 2018 in Sweden. The relationships between physical activity and sleep disturbance with blood *glucose rate of change* were assessed. An interaction term was fitted to determine difference in the relationship between movement and glucose variation, conditional on waking/sleeping. Total movement was inversely related to *glucose rate of change* (p < 0.001, 95% CI (− 0.037, − 0.026)). Stratified analyses showed total physical activity was inversely related to *glucose rate of change* (p < 0.001, 95% CI (− 0.040, − 0.028)), whereas sleep disturbance was not related to *glucose rate of change* (p = 0.07, 95% CI (< − 0.001, 0.013)). The interaction term was positively related to *glucose rate of change* (p < 0.001, 95% CI (0.029, 0.047)). This study provides temporal evidence of a relationship between total movement and glycemic control in pregnancy, which is conditional on time of day. Movement is beneficially related with glycemic control while awake, but not during sleep.

## Introduction

Pregnancy places special demands on energy metabolism, owing to the need for an adequate supply of nutrients, particularly glucose, to the growing fetus. In healthy pregnancies, endocrine adjustments take place progressively from mid- to late-pregnancy. During this period maternal peripheral insulin sensitivity declines and is paralleled by increases in insulin release and blood insulin concentrations^1^. It is not only the peak glucose concentrations that are clinically significant, but so is the variability in glucose concentrations^2,3^, as both may adversely affect the way in which the fetus develops, its birth weight, and its predisposition to obesity and type-2 diabetes mellitus (DM) later in life^4^.

Lifestyle recommendations during pregnancy focus on healthy maternal weight gain through low-to-moderate intensity exercise^5^. Although epidemiological studies have assessed physical activity and risk of gestational DM (GDM), these studies have generally used subjective assessment methods and showed inconclusive results^6^. Cross-sectional studies of accelerometery recorded physical activity in non-diabetic pregnancies demonstrated that total physical activity demonstrates the strongest association with first-phase insulin secretion and estimated insulin sensitivity^7,8^. Moreover, a meta-analysis of both acute and chronic exercise in non-diabetic and diabetic pregnancies reported a dose–response relationship in the reduction of mean blood glucose and fasting blood glucose levels respectively^9^.

Circadian control of diurnal rhythm in glucose metabolism can be disrupted by short sleep duration and fragmented sleep which in turn is associated with increased post-challenge glucose concentrations and reduced insulin sensitivity in studies of non-pregnant women^10–12^. Short sleep duration in pregnancy^13^ and subjectively assessed poor sleep quality have been associated with elevated risk of GDM, but where sleep quality has been objectively assessed, results are inconclusive^14^.

Wearing both CGM and accelerometery devices have been shown to be acceptable to participants^15^. Both devices have been used to assess 24-h movement and glucose variability. Machine learning-based glucose prediction models incorporating accelerometry data have shown slightly improved glucose prediction intervals with higher movement volume^16^.

To our knowledge, no studies are published where both movement and diurnal glucose patterns in pregnancy have been continuously objectively assessed. An improved understanding of the relationship between movement and glucose patterns while awake and sleeping may inform strategies to improve gestational glycemic control. Accordingly, the purpose of this analysis was to assess the relationships of daytime movement (physical activity) and night-time movement (sleep disturbance) with glycemic control during pregnancy, using objective continuous assessment methods.

## Methods

### Study design

This prospective study was conducted at Clinical Studies Sweden—Forum South, Skåne University Hospital, Lund, Sweden from February through June 2018. The women in this cohort participated in the “Diabetes in Pregnancy Methylome Project”. Ethics approval was granted by the regional ethical review board at Lund University, Sweden (Dnr-2016/489; 2019-02846) and all methods were performed in accordance with the relevant guidelines and regulations. This study was strictly observational and participants continued to attend their routine antenatal clinic visits in addition to study visits.

### Recruitment

Midwives recruited pregnant women (20–40 years old; 8–14-weeks’ gestation; of European ancestry, with both parents born in a Scandinavian country) through the Antenatal clinic at the Department of Gynecology and Obstetrics, Skåne University Hospital in Lund, Sweden and the Maternal Health Care centres in the catchment area of the hospital. Exclusion criteria included a past medical history of gastric bypass surgery and/or assisted reproductive technology (e.g. in vitro fertilization or hormonal stimulation in current pregnancy). Current use of certain medications: systemic steroids, antipsychotics, anticonvulsive medications, mood agents, ADHD medications, and/or weight loss medications, and any current major illness that might interfere with active consensual participation or that might confound study results (e.g., psychiatric disorders, type-1 or type-2 diabetes mellitus, polycystic ovary syndrome), were further exclusions. Seventeen nondiabetic pregnant women were enrolled for this study. Following recruitment informed consent was obtained. Eligible women underwent a 7-day run-in period during which compliance to activity and glucose monitoring was assessed; in one case, the run-in period was only 4 days owing to illness, but the participant continued in the study.

### Data collection and derivation of key variables

All data processing was conducted using *R* software version 3.6.1.

### Continuous glucose monitoring

Participants were trained in the use of a Freestyle Libre CGM device (http://freestylelibre.se), including removing and replacing the skin-affixed electrode. Each CGM was calibrated using capillary glucose measurements, according to the manufacturer’s instructions. The CGM device was programmed to record interstitial glucose values, converted to blood glucose in mmol/l, at 15-min intervals, generating 96 recordings per day. Participants downloaded data from the CGM to a handheld glucometer bi-weekly and uploaded to the study server via a secure webportal; these data were subsequently retrieved from the server by study staff.

### Glucose rate of change

The dependent continuous variable, termed ‘glucose rate of change’ was derived from CGM data. It describes blood glucose control as the degree of change in glucose (mmol/l) at timepoint ‘t’ compared with the mmol/l value immediately prior, at timepoint ‘t − 1’, and is expressed as a percentage. This metric is an adaptation of an absolute glucose rate of change over time. Summarised values of the percent glucose rate of change has been used to assess clinical endpoints and was more predictive than metrics based on an absolute glucose rate of change^17^.

$$Glucose\;rate\;of\;change = 100*\left( {Glucose_{t} - Glucose_{t - 1} } \right)/Glucose_{t - 1}$$

The CGM dataset contained duplicate values at timepoints recorded during calibration procedures. The calibrated values were retained for analysis and duplicates were removed. The calculation of glucose rate of change was performed first, by entering consecutive CGM values into the above equation. Secondly, the glucose rate of change values were transformed, using ordered quantile normalization.

### Definition of the main exposures physical activity and sleep

Each woman wore an Axivity AX3 triaxial accelerometer (Axivity Ltd, Newcastle upon Tyne, United Kingdom) on their non-dominant wrist throughout the study. The accelerometer was set to record acceleration at a frequency of 25 Hz (allowing for 34-days of onboard data storage). The accelerometer was recharged every fourth week. Data were obtained from the device at the participants’ convenience (e.g., during study visits, clinic visits or by exchanging the device by mail).

The *R*-implemented tool *GGIR* 2.2-0^18^ was used to read and analyze accelerometery data. This package processes triaxial accelerometery data, removes gravity and sensor noise, determines sustained abnormally high values, identifies wear/non-wear episodes, and provides mean magnitude of dynamic acceleration. The derived gravitational Euclidean norm minus one (ENMO) units (*g*, one *g* = 1000 mg)) are the average magnitudes of dynamic acceleration corrected for gravity; bias and error is reduced by rounding negative ENMO to zero^19–22^. ENMO was subsequently ranked with ‘ties’ allocated to the average of the rank. A participant’s data for a given day was excluded from subsequent analyses if wear time amounted to less than 16-h for that day. Files were excluded if post-calibration error exceeded 0.01 g after reading 72-h of data. Sustained inactivity bouts were determined by setting the z-angle (where the axis is perpendicular to the wrist’s dorsal–ventral direction in the anatomical position) threshold as five degrees and time threshold as five minutes and were required to distinguish between inactivity during waking time and the *GGIR* output *total sleep-period*. The sleep parameters: sleep onset, wakeup onset, total sleep-period, undisturbed sleep time and movement during total sleep-period, which was classed as sleep disturbance (see Supplementary Fig. S1 online), were determined using published algorithms^23^. Accelerometery for each participant was visualised to detect sustained supraphysiological observations, which were supported by autocalibration analyses within *GGIR*^22^.

### Time of day

A two-level factor, was defined denoting a common sleeping and waking period for all the participant. Analyses within *GGIR* produce the timepoints for *sleep onset* and *wakeup onset* for each participant. The distributions of these outputs were visualised; wakeup onset was normally distributed and sleep onset was bimodally distributed.

The *sleep efficiency* variable, for each participant per day, was used as a measure of sleep quality and is a continuous variable derived as the ratio of the *GGIR* assessed sleep parameters undisturbed sleep time, to total sleep-period time of the participant per 24-h period multiplied by 100 and rounded to whole percent^23,24^. As reference, a sleep efficiency ≥ 85%, except in young adults (18–25 years), is considered good sleep quality^24^.

### Anthropometrics

Daily weight in kilograms (kg) was measured using Beurer PS 240 Soft Grip scales (Beurer GmbH, Ulm, Germany) provided to each participant for the duration of the study. The participants recorded daily weight manually in a logbook or by uploading this data directly via a secure web portal. Data on maternal age (years), height (meters, m) and pre-pregnancy weight were collected by trained study staff. Body mass index was calculated as weight (kg) divided by height (m) squared.

Participant weight (kg) and height (m) were entered as recorded after review for implausible values. Participants whose weight changed by > 5 kg from 1 day to the next (< 0.1% of weight entries) had this value replaced by linear interpolation. Participants did not consistently enter daily body weight measurements during the aligned period of physical activity and CGM data, these values (5.1%; 82 weight entries) were imputed by linear interpolation.

### Data alignment

Data recorded by the CGM device and accelerometer had timestamps for each observation. The CGM and accelerometer data were organised in a common data-frame according to timestamp. This ensured that movement (given as a level of mean acceleration) at a given timepoint, could be aligned to a corresponding CGM observation assessed at 15-min intervals. We assessed 2044 days of CGM and 1607 days (79.6%) were aligned with the accelerometery data. Glucose rate of change was subsequently calculated (as described above).

All values from the covariates as well as the outcome variable were aligned to a timestamp. The outcome variable *glucose rate of change* constitutes one observation at a given timestamp. The covariate *movement* likewise constitutes one observation per timestamp. The covariates *weight* and *sleep efficiency* were assessed once per 24-h; since each observation is assigned one timestamp, the values of these covariates were repeated for each timestamp of that corresponding day. The covariates *height* and *age* were constant; these observations were repeated in all timestamps. The covariate *time of day* was coded as a dummy variable, where the reference, *waking period, was assigned the value* “0” and the *sleeping period* was assigned the value “1”. The value “0” was aligned to all timestamps from 06:55 to 22:08 and “1” was aligned to all timestamps from 22:08 to 06:55.

### Statistical analysis

The main statistical analyses were performed using linear mixed effects models within the *NLME* package^25^, with *participant* as a random effect (for details see Supplement online). Models were created where each random effect term had its own intercept but shared coefficients for the fixed effects. Because the number of data-points for each data-series varied by participant, restricted maximum likelihood estimations were included to distinguish between variance components.

The models were constructed sequentially. The outcome (dependent) variable *glucose rate of change* was regressed with physical activity grouped by participant (not shown, as this model yielded the same results as subsequent models). The fixed-effects covariates (exposures), *weight*, *height*, *age*, *sleep efficiency* and *time of day* were added stepwise with each subsequent model.

The first linear mixed effects model assessed *movement*, *weight*, *height* and *sleep efficiency*. A second model was constructed as the first, but adjusted for *time of day*; *time of day* was a potential effect modifier and potential confounder for total movement. Data were subsequently stratified by *waking period* and *sleeping period*, and marginal effects models were run for each period separately; the regressions’ estimates for *movement* in these models were qualitatively different. The final full model was constructed as the second model, but with an interaction term of *time of day* and *movement*; the interaction of *time of day* and *movement* was modelled. Given that glycemic control in pregnancy is known to vary by adiposity^26^ and age^27^, the final model included *weight* adjusted for *height* and *age*.

The variance of the mixed effects models’ residuals increased with higher fitted values; this was controlled for by transforming the glucose rate of change values using ordered quantile normalization, ranking movement data and weighted regression (by specifying that residual variance should be proportional to the movement data’s variance structure). The variance structure of the within group errors, was modelled as the power of the absolute value of ranked movement data^28^ (for details see Supplement online). The full model was optimised by modelling the variance structure of movement data stratified by the factor time of day. This selection was made after running an analysis of variance to compare full models weighted by an unstratified and stratified variance function by time of day.

The aligned data was analysed to determine if data was preferentially collected during *waking* or *sleeping periods* or on specific days of the week. Summary statistics are presented as medians with 1st and 3rd quartile (Q1–Q3). Given the hypothesis-driven nature of these analyses, and the greater than null priors this affords, the threshold for statistical significance was set to < 0.05. Linear mixed effects model results are given as measures of association and 95% confidence intervals (95% CI).

## Results

### Descriptive statistics

**Figure 1:**
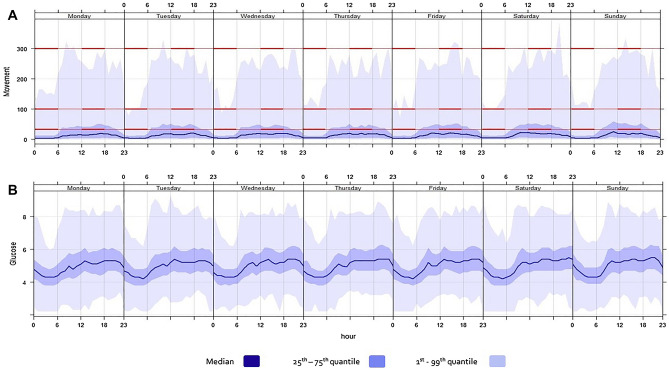
Timeseries of blood glucose and movement: Timeseries of the study cohort—movement and blood glucose values summarised by quantiles: (**A**) Movement (mg) and (**B**) Glucose (mmol/l), exhibiting the diurnal pattern of the entire time series summarised into one week. Nocturnal quantiles of movement and glucose < daytime quantiles. The red lines indicate thresholds for movement intensity. Movement < 30 mg was classed as inactivity (sitting), light intensity movement is ≥30 mg and < 100 mg (slow walks, 3 km/h), moderate intensity movement is ≥ 100 mg and < 400 mg (fast walks, 5 km/h). Vigorous intensity movement ≥400 mg (running, 8 km/h) < 1 quantile of total movement.

Of the 17 enrolled women, one withdrew early in the study, a second withdrew after 2 months (available data for this participant were included in the analysis with her consent), and insufficient activity and glucose monitoring data were available for a third woman (excluding her from this analysis). The total amount of aligned CGM and accelerometery data for the 15 women totalled 1607 days-worth of data. In this cohort, four women had a family history of DM (any type), seven had a family history of cardiovascular disease and eight of hypertension. Cohort characteristics and summary values for blood glucose, glucose rate of change and movement for all women are shown in Table 1. Figure 1 visualises the distribution of diurnal blood glucose and movement and approximated intensities based on levels of energy metabolism as described elsewhere^29,30^. The summary values for time of day for all women were a waking period from 06:55 to 22:08 and sleeping period from 22:08 to 06:55. A total of 63.5% of data was sited under the waking period and 36.6% under the sleeping period. The waking period duration was 64.1% (15.4 h) and the sleeping period 35.9% (8.6 h) of 24 h. The data were evenly distributed between each weekday and time of day per woman. The cohort’s summary values are presented in Supplementary Table S1 online.

**Table 1:** Study cohort characteristics and values for blood glucose (mmol/l), glucose rate of change (%) and movement (mg) over 24 h and for waking and sleeping periods. Presented as median with 1st and 3rd quartiles (Q1–Q3).

	Median	Q1–Q3
Age (years)	29.5	(27.9, 32.3)
Pre-preg. BMI^a^ (kg/m^2^)	22.7	(21.3, 25.7)
Weight gain (kg)	15.5	(10.6, 17.6)
GA-Start^b^ (weeks)	17.7	(14.5, 20.9)
GA-End^c^ (weeks)	38.9	(38.0, 39.6)
Days^d^	105	(94, 122)
Sleep Efficiency (%)	91	(88, 94)
**Glucose**		
24 h	4.9	(4.3, 5.7)
Waking period	5.2	(4.5, 5.9)
Sleeping period	4.5	(4.0, 5.2)
**Glucose rate of change**		
24 h	0.0	(− 4.5, 4.3)
Waking period	0.0	(-4.9, 5.2)
Sleeping period	0.0	(− 4.2, 2.6)
**Movement**		
24 h	10.0	(1.6, 32.0)
Waking period	16.2	(3.6, 43.3)
Sleeping period	4.4	(0.1, 13.8)

^a^Pre-pregnancy body mass index.

Gestational age at the start^b^ and end^c^ of the aligned movement and glucose rate of change data.

^d^Days of aligned movement and glucose rate of change data.

### Linear mixed effects models

#### The relationship of weight and maternal age with glucose rate of change

Increasing weight per kg (adjusted by height) in pregnancy has a negative relationship with glucose rate of change, but only statistically significant during sleeping period (estimate − 0.159% per quartile weight increase, 66 kg, 95% CI (− 0.259, − 0.060)) (Table 3). Maternal age did not have a statistically significant relationship with glucose rate of change. Participant weight (adjusted by height) and age did not influence the relationship of total physical activity with glucose rate of change.

#### Interaction of time of day and movement on glucose rate of change

Total movement has a negative relationship with glucose rate of change. Comparing the first and second models (Table 2), results indicate that the effect of total movement on glucose rate of change was attenuated by time of day. An increment of total movement by one quartile, 1.6 mg, was associated with a decrease in glucose rate of change of 0.032% (95% CI (− 0.037, − 0.026)) in the full model (Table 4). Sleeping period had a negative relationship with glucose rate of change compared to waking period. The relationship of sleeping period to glucose rate of change is less than that of the waking period by 0.166% (95% CI (− 0.185, − 0.146)) in the full model (Table 4).

**Table 2:** Relationships of movement, weight, height, age, sleep efficiency with glucose rate of change (%) in pregnancy, unadjusted and adjusted for time of day (95% CI) from linear mixed effects models. Regression coefficients are given by the 1st quartile unit increases for movement, weight, height, age and sleep efficiency.

Covariates	Estimate	95% CI
**First linear mixed model**^**1**^		
Movement^a^	− 0.007	(− 0.011, − 0.003)**
Weight^b^	− 0.020	(− 0.054, 0.015)
Height^c^	0.112	(− 0.133, 0.359)
Age^d^	0.034	(− 0.031, 0.100)
SE^e^	− 0.242	(− 4.513, 4.030)
N^g^	154,262	
**Second linear mixed model**^**2**^		
Movement^a^	− 0.019	(− 0.024, − 0.015)**
Weight^b^	− 0.022	(− 0.057, 0.014)
Height^c^	0.115	(− 0.141. 0.372)
Age^d^	0.040	(− 0.029, 0.107)
SE^e^	− 0.371	(− 4.645, 3.898)
TD^f^	− 0.100	(− 0.111, − 0.089)**
N^g^	154,262	

The 1^st^ quartiles: Movement = 1.6* mg*. Weight = 66 kg. Height = 1.63 m. Age = 27.9 years. Sleep efficiency = 88%.

^1^First linear mixed effects model: ^a^Movement (*mg*), ^b^Weight (kg), ^c^Height (m), ^d^Age (years), ^e^Sleep efficiency (%).

^2^As the first linear mixed effects model but additionally adjusted for ^f^Time of day (2-level factor, sleeping period and waking period, used as reference).

^g^Number of observations.

**Statistically significant results.

Two further models were run, stratified into a waking period and a sleeping period (Table 3). During the waking period, the relationship of total physical activity (i.e. movement during waking period) with glucose rate of change remained inverse. An increment of total physical activity by one quartile, 3.6 mg, is associated with a decrease in glucose rate of change of 0.034% (95% CI (− 0.040, − 0.028)) (Table 3). Total sleep movement had a positive relationship with glucose rate of change during the sleeping period, though this was not statistically significant (95% CI (< − 0.001, 0.013)) (Table 3).

**Table 3:** Relationships of movement, weight, height, age, sleep efficiency with glucose rate of change (%) in pregnancy in stratified analyses by waking period and sleeping period (95% CI). Regression coefficients are given by the 1st quartile unit increases for movement, weight, height, age and sleep efficiency.

Covariates	Estimate	95% CI
**Waking period**^**1**^		
Movement^a^	− 0.034	(− 0.040, − 0.028)**
Weight^b^	− 0.003	(− 0.058, 0.052)
Height^c^	− 0.052	(− 0.479, 0.377)
Age^d^	0.007	(− 0.119, 0.132)
SE^e^	0.133	(− 5.575, 5.839)
N^f^	98,912	
**Sleeping period**^**2**^		
Movement^a^	0.006	(< − 0.001, 0.013)
Weight^b^	− 0.159	(− 0.259, − 0.060)**
Height^c^	0.792	(− 0.051, 1.630)
Age^d^	0.135	(− 0.096, 0.366)
SE^e^	− 1.853	(− 8.165, 4.460)
N^f^	55,350	

The 1^st^ quartiles: Movement (waking period) = 3.6* mg*. Movement (sleeping period) = 0.1* mg*. Weight = 66 kg. Height = 1.63 m. Age = 27.9 years. Sleep efficiency = 88%.

Linear mixed effects models stratified for waking period^1^ and sleeping period^2^: ^a^Movement (*mg*), ^b^Weight (kg), ^c^Height (m), ^d^Age (years), ^e^Sleep efficiency (%).

^f^Number of observations.

**Statistically significant results.

The relationship of total movement and glucose rate of change varies by time of day as indicated by the significant interaction between total movement*time of day (with waking period as reference) (Table 4). The relationship of one quartile total movement, 1.6 mg, and glucose rate of change conditioned by sleeping period is greater than one quartile total movement conditioned by waking period by 0.038% (95% CI (0.029, 0.047)) (Table 4). Sleep efficiency was not related with glucose rate of change (Table 4).

**Table 4:** Relationships of movement, weight, height, age, sleep efficiency, time of day and interaction term (movement & time of day) with glucose rate of change (%) in pregnancy (95% CI). Regression coefficients are given by the 1st quartile unit increases for movement, weight, height, age and sleep efficiency.

Covariates	Estimate	95% CI
**Full linear mixed effects model**^**1**^		
Movement^a^	− 0.032	(− 0.037, − 0.026)**
Weight^b^	− 0.027	(− 0.066, 0.011)
Height^c^	0.143	(− 0.137, 0.422)
Age^d^	0.046	(− 0.028, 0.121)
SE^e^	− 0.506	(− 4.732, 3.723)
TD^f^	− 0.166	(− 0.185, − 0.146)**
TD*M^g^	0.038	(0.029, 0.047)**
N^h^	154,262	

The 1st quartiles for movement, weight, height, age and sleep efficiency are as follows: Movement = 1.6 mg. Weight = 66 kg. Height = 1.63 m. Age = 27.9 years. Sleep efficiency = 88%.

^1^Full linear mixed effects model: ^a^Movement (*mg*), ^b^Weight (kg), ^c^Height (m), ^d^Age (years), ^e^Sleep efficiency (%), ^f^Time of day (2-level factor, sleeping period and waking period, as reference).

^g^Interaction term of time of day and movement.

^h^Number of observations.

**Statistically significant results.

## Discussion

In this study, we show a relationship between total movement and improved glycemic control in pregnancy and that this association is contingent on whether movement occurs during waking or sleeping periods. To our knowledge this study is unique, owing to data continuously collected over 20 gestational weeks, with a median of 105-days (Q1–Q3: 94, 122) of complete data throughout pregnancy; this allows temporality between the relationship of total movement and glycemic control to be defined, thereby extending previous studies. A stratified analysis showed that movement while awake (physical activity) was strongly related with improved glycemic control, whereas movement during sleeping periods tended to associate with poorer glycemic control. This study could not however demonstrate a relationship between sleep quality, assessed as *sleep efficiency*, and glycemic control, possibly because this variable is less sensitive than night-time movement quantified as acceleration.

Our results relating to total physical activity are in concordance with previous studies of exercise and total physical activity that show a beneficial association with glycemic control^7,8^ and reduced risk of GDM^6,9^. Studies of low intensity physical activities in the form of habitual walking in pregnant women without^31^ and with^32^ diabetes have demonstrated decrements in non-fasting glucose levels. However, few published studies have utilised objective assessments of overall movement or CGM, without which it is challenging to determine the relationship of non-structured physical activities with blood glucose levels. Thus, the current study adds to the existing literature by showing that overall movement, including light activities, is likely to help maintain glucose control in pregnancy.

Published studies show that self-reported and objectively assessed sleep duration is associated with blood glucose levels^13,33^. A limitation in these studies and our study is that physical activity related energy expenditure (EE) during pregnancy is not controlled for. There may be associations between energy and/or motivation to perform leisure associated physical activity. A previous study used a validated survey to assess for an association between sleep, leisure associated physical activity EE and glucose tolerance testing and reported that shorter sleep duration is associated with greater fasting glucose levels independent of EE during early-mid pregnancy^34^. In this study, the relationship between movement and glycemic control was conditional on waking or sleeping. While waking movement (physical activity) is inversely related with glucose regulation, movement whilst asleep was positively associated with *glucose rate of change*, albeit at a nominal level of statistical significance. A fasting period coincides with night-time sleep, where metabolism relies less on glucose and more on free fatty acid oxidation^35^. The reduction of this fasting period has been associated with increased fasting glucose, insulin resistance, and excessive weight gain during gestation^36^. Hepatic glucose production declines during sleep and there may be an anticipatory increase just before breakfast, although not all studies have shown this^37.^ While this interaction between movement and time of day may appear counterintuitive, the movement assessed during sleeping periods is plausibly associated with events that disrupt the beneficial effects of sleep on glycemic control^37^. In this light, results are concordant with earlier studies showing a relationship between sleep deprivation and poor glycemic control as well as a greater risk of GDM^13,14^.

Weight had a negative relationship with glucose rate of change during sleeping period and may be a false positive. Participants with greater weight could have positioning difficulties, increased musculoskeletal discomfort or sleep-disordered breathing, which might contribute to sleep disturbance. Weight adjusted by height was likely a poor estimate for adiposity. Greater adiposity has been associated with higher nocturnal (and daytime) glycemic profiles in non-diabetic pregnancies^38^.

Sleep efficiency was not related with glucose rate of change in this study. A meta-analysis of studies using subjective assessments of sleep quality revealed an association with GDM^14^. However, of the eight published studies investigating sleep quality, only three used objective measures and these were inconclusive^39–41^. A direct comparison between these earlier studies and ours cannot be made, as two of the studies primarily investigated sleep-disordered breathing^39^ and obstructive sleep apnoea^40^ using polysomnography, while the third study assessed the association between sleep fragmentation index and GDM^41^.

Maternal age did not have a relationship with glycemic control in this study despite being a well-known risk factor for glucose intolerance^27^; the small sample size and low variation of participants’ age were likely contributing reasons.

## Strengths and limitations

A strength of this study was the use of wrist-worn accelerometery to objectively assess movement over a 24-h period including sleep parameters. The algorithm used to assess sleep parameters was validated against sleep diaries and polysomnography^23^. Although the method used here to assess sleep quality has the advantage of being objective in nature it is not able to determine sleep phase. Thus, the results of this study should be considered with some caution, as sleep disturbance may not adequately reflect the quality of the periods when the participant was sleeping and remained still. The use of the sleep efficiency metric has its limitations, not least that the threshold for good quality sleep is based purely on expert opinion^24^. This metric can be calculated using assessments by both objective and subjective methods, which makes comparisons between studies difficult.

Field staff were blinded to the results and participants did not have access to the accelerometery data. CGM calibration is a necessary component of the protocol, yet blinding participants or clinicians to CGM data would be unethical as it would delay action on potentially abnormal results. Neither a sleep log nor log of potential shift work was kept, but *GGIR* output on sleeping period time primarily spanned during evenings and nights. Food diaries were not obtained continuously throughout the study period owing to burden on study participants. Food consumption may coincide with activity bouts and impact blood glucose variation^42^. Therefore, distinguishing the impact of movement on blood glucose variation at and around meals is not possible in this study. Nevertheless, the overall pattern of movement and glucose variability as described here is likely to generalize to other pregnancy cohorts, as the coincidence of mealtimes and reduced movement is a generic factor, with people generally moving less when consuming a meal”.

The cohort used in these analyses is relatively small. However, the large volume of data collected within each participant, and the analysis of these data using linear mixed-effects models, helps overcome some of the limitations of the small study sample. Indeed, the associations between movement and glycemic control are detectable in this small cohort because the data are high-resolution and relatively precise, and also because the associations are of relatively large magnitude. Some of the drivers of movement during sleep differ from those during daytime physical activity. The sleep algorithm used in this study cannot determine whether the movement during the sleeping period is caused by a primary sleep disorder or an unrelated pathology during pregnancy, such as mechanical back pain. Yet it is a cost effective and pragmatic method of identifying pregnant women at risk of poorer nocturnal blood glucose control. Given the popularity of wrist worn accelerometers (e.g. Fitbit), our findings may help users of those devices, or their health providers, better understand the impact of disturbed sleep on glucose regulation.

## Conclusion

This study demonstrates that while movement during daytime is associated with better glycemic control, night-time movement is not. This illustrates the importance of non-structured everyday physical activities and high-quality sleep in the regulation of blood glucose in pregnancy. Future studies are required to understand the specific nature of sleep disturbances that affect nocturnal blood glucose regulation and how these relate to pregnancy outcomes, particularly during GDM.

## Supplementary Information

Supplementary Information.

## Data Availability

Summary level data may be accessible upon request, but individual level data are not available owing to consenting constraints.
