# Supplementary material for: A prospective study of the relationships between movement and glycemic control during day and night in pregnancy
Source: Sci Rep. 2021 Dec 13;11:23911. doi: 10.1038/s41598-021-03257-0 (PMC8668873; doi:10.1038/s41598-021-03257-0)
Supplement: Supplementary file 1 — Supplementary Information. [file 41598_2021_3257_MOESM1_ESM.pdf]

A prospective study of the relationships between movement and glycemic control during day and night in pregnancy

Authors

Masoud Behravesh[1], Juan Fernandez-Tajes[1], Angela C Estampador[1], Tibor V Varga[1,2], Ómar S Gunnarsson[3,4], Helena Strevens[3], Simon Timpka[3,4], Paul W Franks[1,5]

1. Genetic & Molecular Epidemiology Unit, Department of Clinical Sciences, Lund University, Malmö, Sweden
2. Section of Epidemiology, Department of Public Health, University of Copenhagen, Copenhagen, Denmark
3. Department of Obstetrics and Gynecology, Skåne University Hospital, Malmö and Lund, Sweden
4. Perinatal and Cardiovascular Epidemiology, Department of Clinical Sciences, Lund University, Malmö, Sweden
5. Harvard TH Chan School of Public Health, Boston, MA, USA

Supplementary information

Statistical Analysis

Linear mixed effects model

$$Y_{ij} = \beta_{0i} + X_{ij}\beta_{ij} + \alpha_{ij}b_i + \epsilon_{ij},$$

$$i = 1, 2 \dots (number\ of\ participants)\ and\ j = 1,2 \dots (number\ of\ observations)$$

The predicted outcome variable of the model, is Y_{ij}, the jth observation in i^{th} group, i.e. each participant, and is conditional on the variation of the sampled population; X_{ij} is the covariate of this response for each participant. The fixed effects coefficients are the regression slopes, β_{ij} , and are shared by all participants and β_{0i}, the intersect, is separate for each participant. The random effect covariate is α_{ij} for j^{th} observation in i^{th} group, each participant, where b_i is the random effect coefficient for i, each participant. The error term, ϵ_{ij}, models the error for observations j^{th} observation in i^{th} group and between groups[1].

The model was used to regress *glucose rate of change* by the within-group fixed-effects covariates *movement*, daily *weight*, *height*, *age*, daily *sleep efficiency*, *time of day* and an interaction term of time of day with overall movement at corresponding time-points. This is premised on the notion of statistical interaction (i.e. that the relationship between movement and blood glucose is "conditional on time of day").

Modelling the Variance structure of the linear mixed effects models

$$Var(\varepsilon_{ij}) = \sigma^2 |v_{ij}|^{2\delta},$$

$$i = 1, 2 \ldots (number\ of\ participants)\ and\ j = 1,2 \ldots (number\ of\ movement\ values)$$

$Var(\varepsilon_{ij})$ is the variance structure of within group errors, σ^2 is the variance modelled by v_{ij}, a vector of variance covariates, of jth in i^{th} participant, and δ is a vector of variance parameters. The variance weights were constrained to 1. The variance structure of movement data stratified by the factor time of day is given as, where s_{ij} is the stratification variable for time of day.

$$Var(\varepsilon_{ij}) = \sigma^2 |v_{ij}|^{2\delta s_{ij}},$$

$$i = 1, 2 \ldots (number\ of\ participants)\ and\ j = 1, 2 \ldots (number\ of\ movement\ values)$$

Assessment of data distribution by day of the week and time of day.

The percentage of observations per participant during *waking* and *sleeping period* was calculated by dividing the number of observations in each period by total observations in the timeseries and multiplying by 100. The percentage of observations by weekday per participant was calculated by dividing the number of observations on a specific weekday by the total number of observations in the timeseries multiplied by 100.

References:

1 Pinheiro, J. C. & Bates, D. M. *Mixed-effects models in S and S-PLUS.*, 201-26 (Springer, 2000).

Supplemental Tables/Figures

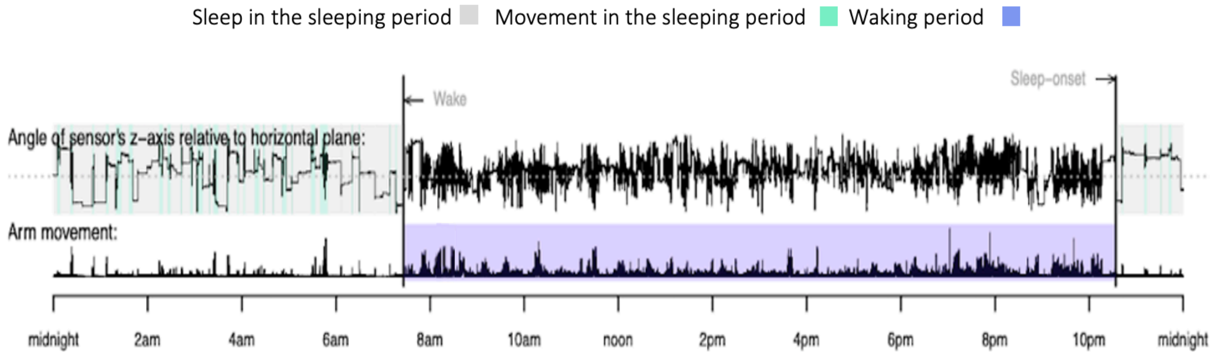

Figure S1 Sleep analysis: Sleeping period is the period indicated by arrows from *sleep-onset* to *wake* (waking period is indicated by blue). Grey indicates undisturbed sleep and green indicates movement in the sleeping period. Once the threshold angle and threshold duration of a person's arm relative to the z-axis is exceeded, arm movement in the sleeping period is classed as physical activity.

Table S1
Study cohort distribution (%) of aligned **movement** and **glucose rate of change** data by weekday and **time of day**. Presented as median with 1st and 3rd quantiles (Q1-Q3).

Weekday	Median	Q1-Q3
Sunday	14.3	(14.0, 14.5)
Monday	14.2	(13.7, 14.8)
Tuesday	14.4	(14.1, 14.7)
Wednesday	14.3	(14.0, 14.5)
Thursday	14.3	(14.0, 14.7)
Friday	14.4	(14.0, 14.6)
Saturday	14.4	(14.2, 14.7)
Waking period	63.8	(63.3, 64.7)
Sleeping period	36.2	(35.4, 36.8)
